# The prediction of asymptomatic carotid atherosclerosis with electronic health records: a comparative study of six machine learning models

**DOI:** 10.1186/s12911-021-01480-3

**Published:** 2021-04-05

**Authors:** Jiaxin Fan, Mengying Chen, Jian Luo, Shusen Yang, Jinming Shi, Qingling Yao, Xiaodong Zhang, Shuang Du, Huiyang Qu, Yuxuan Cheng, Shuyin Ma, Meijuan Zhang, Xi Xu, Qian Wang, Shuqin Zhan

**Affiliations:** 1 Department of Neurology, The Second Affiliated Hospital of Xi’an Jiaotong University, No. 157 West Five Road, Xi’an, 710004 Shaanxi China; 2 Faculty of Electronic and Information Engineering, Xi’an Jiaotong University, Xi’an, China; 3 School of Mathematics and Statistics, Xi’an Jiaotong University, Xi’an, China; 4 Department of Health Management, The Second Affiliated Hospital of Xi’an Jiaotong University, Xi’an, China

**Keywords:** Machine learning, Asymptomatic carotid atherosclerosis, Electronic health records, Prediction

## Abstract

**Background:**

Screening carotid B-mode ultrasonography is a frequently used method to detect subjects with carotid atherosclerosis (CAS). Due to the asymptomatic progression of most CAS patients, early identification is challenging for clinicians, and it may trigger ischemic stroke. Recently, machine learning has shown a strong ability to classify data and a potential for prediction in the medical field. The combined use of machine learning and the electronic health records of patients could provide clinicians with a more convenient and precise method to identify asymptomatic CAS.

**Methods:**

Retrospective cohort study using routine clinical data of medical check-up subjects from April 19, 2010 to November 15, 2019. Six machine learning models (logistic regression [LR], random forest [RF], decision tree [DT], eXtreme Gradient Boosting [XGB], Gaussian Naïve Bayes [GNB], and K-Nearest Neighbour [KNN]) were used to predict asymptomatic CAS and compared their predictability in terms of the area under the receiver operating characteristic curve (AUCROC), accuracy (ACC), and F1 score (F1).

**Results:**

Of the 18,441 subjects, 6553 were diagnosed with asymptomatic CAS. Compared to DT (AUCROC 0.628, ACC 65.4%, and F1 52.5%), the other five models improved prediction: KNN + 7.6% (0.704, 68.8%, and 50.9%, respectively), GNB + 12.5% (0.753, 67.0%, and 46.8%, respectively), XGB + 16.0% (0.788, 73.4%, and 55.7%, respectively), RF + 16.6% (0.794, 74.5%, and 56.8%, respectively) and LR + 18.1% (0.809, 74.7%, and 59.9%, respectively). The highest achieving model, LR predicted 1045/1966 cases (sensitivity 53.2%) and 3088/3566 non-cases (specificity 86.6%). A tenfold cross-validation scheme further verified the predictive ability of the LR.

**Conclusions:**

Among machine learning models, LR showed optimal performance in predicting asymptomatic CAS. Our findings set the stage for an early automatic alarming system, allowing a more precise allocation of CAS prevention measures to individuals probably to benefit most.

**Supplementary Information:**

The online version contains supplementary material available at 10.1186/s12911-021-01480-3.

## Background

Carotid atherosclerosis (CAS) is a complex disease [1], which reflects cerebral atherosclerosis to a certain extent and can trigger ischemic stroke. The atherosclerotic process usually originates early in life and the condition remains asymptomatic for several decades. The standardised prevalence of asymptomatic CAS in China is 36.2% [2]. One of the key measures to delay the development of asymptomatic CAS into symptomatic CAS and cerebrovascular events is to identify apparently healthy individuals with risk factors and control them as early as possible [3]. However, the early diagnosis of asymptomatic individuals remains a challenge for clinicians.

Machine learning can effectively configure multimodal data and achieve a precise predictive ability to assess diagnostic and prognostic outcomes [4]. Medicine is undergoing an electronic revolution, more and more electronic medical records are available, laying the cornerstone for personalised medicine mediated by computer technology. Mounting studies [5–8] have shown that machine learning yields satisfactory results in biomedicine. One meta-analysis [9] revealed that the diagnostic performance of deep learning models was comparable to that of healthcare professionals. Driven by market forces and a strong public interest, such machine- learning-based predictive tools require rapid development.

In neurology, machine learning has been increasingly applied in disease diagnosis, treatment, and outcome prediction [10, 11]. To date, no study has applied machine learning algorithms to predict asymptomatic CAS. In this context, we used and compared multiple machine learning models to predict asymptomatic CAS subjects using electronic health records.

## Methods

### Study design and data collection

**Fig. 1:**
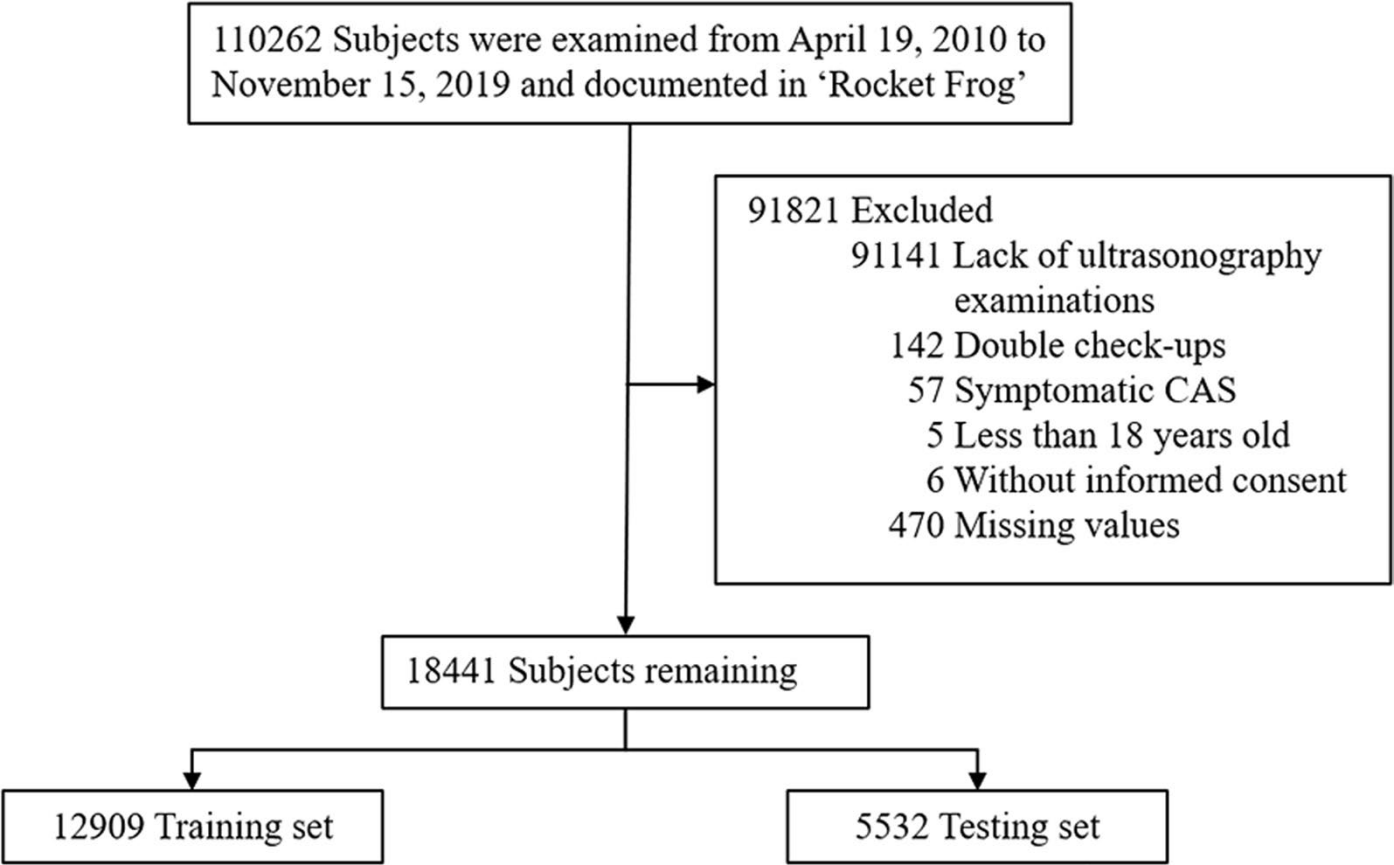
Flowchart illustrating sample selection. (CAS, carotid atherosclerosis)

Electronic health records of medical check-up subjects were retrospectively extracted from the Department of Health Management at The Second Affiliated Hospital of Xi'an Jiaotong University from April 19, 2010 to November 15, 2019. Inclusion criteria: (1) Data documented in "Rocket Frog" (Beijing, China), an electronic health record system; (2) Age ≥ 18 years old; (3) Lack of symptoms, such as limb weakness, aphasia, transient monocular blindness, dizziness, crooked mouth, dysphagia, and coma; (4) Complete carotid B-mode ultrasonography examination; (5) No missing values; (6) Provision of informed consent (refer to the Ethics approval and consent to participate and Statement sections for more details). Subjects were excluded if they did not meet the above criteria. If the subject had completed more than one check-up, the most recent report was included, as this would be more closely related to the physical condition of people. The sample selection process is summarised in Fig. 1. To ensure data accuracy, data were collected independently by two clinicians. If the data were not identical after assessment using the assert_frame_equal module from pandas, a third clinician would reconfirm the data.

CAS was diagnosed by carotid B-mode ultrasonography [12], which was defined as a carotid intima-media thickness of 1.0 mm or greater, or plaque formation. We used a carotid ultrasound machine (Preirus, Hitachi, Tokyo, Japan) with a probe (5–10 MHz; L7-3, Tokyo, Japan). To maintain a balance between positive and negative cases, the participants were randomly divided into a training set (70%) and a testing set (30%), as stratified by CAS. Briefly, we first separated the positive and negative cases from the entire dataset. Then, 70% of the positive cases were randomly assigned to the training set and the remaining 30% were assigned to the testing set. The division of negative cases was the same as that in positive cases.

### Features preprocessing

Before implementing the models, we first preprocessed candidate features on the complete dataset, which represented the personal characteristics, vital signs, co-morbid conditions, laboratory values, and physical examinations (see Additional file 1: Table 1 for more details). Using asymptomatic CAS as a dependent variable, we performed binary logistic regression, a widely and traditionally used method in the medical and biological sciences [13] to select statistically significant features. Categorical features were encoded as binary input features and continuous features were scaled to span the range [0, 1].

**Table 1:** The characteristics of 18,441 participants

Feature	Training set (n = 12,909)	Testing set (n = 5532)	Total population (n = 18,441)
*Characteristics*			
Age	50.88 (19, 96)	50.81 (18, 93)	50.86 (18, 96)
Age subgroup, y			
18–64	11,170 (86.6)	4815 (87.0)	15,991 (86.7)
> 64	1733 (13.4)	717 (13.0)	2450 (13.3)
Gender (male)	7738 (59.9)	3297 (59.6)	11,035 (59.8)
SBP, mmHg	128.69 ± 17.76	128.04 ± 17.43	128.49 ± 17.66
Heart rate, beats/min	75.71 ± 7.80	75.48 ± 7.93	75.64 ± 7.84
Pulse, beats/min	77.40 ± 10.88	76.92 ± 10.94	77.25 ± 10.90
Waistline, cm	83.66 ± 10.10	83.70 ± 9.89	83.67 ± 10.04
*Co-morbid conditions*			
Hypertension	1502 (11.6)	868 (15.7)	2370 (12.9)
Diabetes mellitus	524 (4.1)	326 (5.9)	850 (4.6)
Hyperlipidemia	177 (1.4)	210 (3.8)	387 (2.1)
Family history	287 (2.2)	235 (4.2)	522 (2.8)
Ever-smoker	563 (4.4)	284 (5.1)	847 (4.6)
*Laboratory values*			
Glucose, mmol/L	5.42 ± 1.36	5.31 ± 1.36	5.39 ± 1.36
HDL, mmol/L	1.23 ± 0.21	1.21 ± 0.25	1.22 ± 0.22
TC, mmol/L	4.40 ± 0.62	4.40 ± 0.80	4.40 ± 0.68
Total protein, g/L	68.89 ± 4.21	69.15 ± 3.68	68.96 ± 4.06
Albumin, g/L	44.30 ± 2.78	44.21 ± 2.40	44.27 ± 2.67
Albumin/Globulin	1.84 ± 0.28	1.82 ± 0.29	1.84 ± 0.28
γ-GLT, U/L	27.33 ± 26.86	27.12 ± 25.18	27.27 ± 26.37
Platelets, 10^9/L	217.36 ± 58.42	218.57 ± 57.25	217.72 ± 58.07
*Carotid atherosclerosis*			
Yes	4587 (35.5)	1966 (35.5)	6553 (35.5)
Age 18–64 y	3246 (25.2)	1418 (25.6)	4670 (25.3)
Age > 64 y	1335 (10.3)	548 (9.9)	1883 (10.2)
No	8322 (64.5)	3566 (64.5)	11,888 (64.5)
Age 18–64 y	7924 (61.4)	3397 (61.4)	11,321 (61.4)
Age > 64 y	398 (3.1)	169 (3.1)	567 (3.1)

Categorical features represented as frequency (%). Continuous features represented as median ± SD, except age, which was median (minimum, maximum). (SBP, systolic blood pressure; HDL, high density lipoprotein; TC, total cholesterol; γ-GLT, γ-glutamyl transpeptidase)

### Models comparison for binary classification problem

After that, we used six state-of-the-art machine learning algorithms to predict the probability of a binary outcome (asymptomatic CAS or non-asymptomatic CAS): logistic regression (LR), random forest (RF), decision tree (DT), eXtreme Gradient Boosting (XGB), Gaussian Naïve Bayes (GNB), K-Nearest Neighbour (KNN), because they are touted as currently widely and successfully classifiers for clinical data [14–16].

LR, a generalised linear regression analysis model, is used to predict a categorical dependent variable based on one or more predictor (independent) variables. That is, it is used to estimate the expectation values of each parameter in a qualitative response model [17]. This algorithm was implemented using the LogisticRegression module of Scikit-Learn. RF is a recursive method using randomization and bagging to increase the variance of ensemble trees. The outcome is directly related to the number of trees in a forest [18]. The higher the number of trees, the more exact the results obtained. We implemented it using Scikit-Learn's RandomForestClassifier module. DT [19] is used for discriminant analysis and constructed by recursive partition, whose information theory includes ID3, C4.5 and CART. ID3 cannot handle continuous data and when processing continuous attribute data, the efficiency of C4.5 is easily negatively affected by data discretization [20]. In the process of constructing the classification tree, CART uses the discretized continuous attribute derived from the Ginigain minimum of the selection criterion as the cut-off point, and the dichotomy can simplify the DT and improve its efficiency [21]. In this study, we used the CART algorithm to construct an asymptomatic CAS prediction model. XGB [7] is an optimized distributed gradient boosting library designed to be excellently scalable and highly efficient. As a modified algorithm based on the traditional gradient boosting decision tree, XGB reduces the risk of overfitting by adding regular terms and directly uses the first and the two order derivatives of the loss function [22, 23]. This algorithm was implemented using Scikit-Learn's XGBClassifier module. Naïve Bayes (NB) [24] applies Bayes' theorem with the "naive" assumption of independence between every set of features, meaning that all features contribute independently to the probability of the target outcome [25]. When the likelihood of features is presumed to be Gaussian, GNB is obtained [26]. This algorithm was implemented using Scikit-Learn's GaussianNB module. KNN [27] is based on a wealth of information among the k-closest neighbours of existing data to predict new data. In fact, it does not construct a model to predict asymptomatic CAS. Instead, the prediction is based on the largest proportion of the k-closest point, so it is often called a lazy classifier [28]. This algorithm was implemented using Scikit-Learn's KNeighborsClassifier module.

Since the values of hyperparameters must be set in advance and cannot be automatically obtained from data [11], tuning parameters are critical and specific for each model. GridSearchCV, which is implemented by estimators, is a traditional way for hyperparameter adjustment in any classification method. Consistent with the study reported by Puneet et al. [29], we optimized hyperparameters using Scikit-Learn's GridSearchCV module and fitted them to the training set without a specific validation set. Briefly, before GridSearchCV performed all necessary model fitting and outperformed the best hyperparameters, a dictionary was defined to store the hyperparameters which needed to be searched first. Fitting the GridSearchCV object not only searched for the best hyperparameters, but also obtained a new training model which automatically fitted the best cross-validation performance hyperparameters of all training sets [30]. After obtaining the optimal hyperparameter combination of each algorithm, we evaluated the model using a 30% hold-out testing set.

Considering that 30% hold-out validation may also suffer from overfitting, we performed tenfold cross-validation scheme to avoid this problem [31]. To achieve this, first, the data were partitioned into 10-equal parts. The model was trained on 9 parts and leaving 1 part for testing. This process was repeated 10 folds while changing the test part one-by-one until testing was performed on all the 10 parts.

### Predictive performance measurements

Several metrics were used to evaluate performance: accuracy, F1 score, specificity, precision, recall and we visualized the area under the receiver operating characteristic curve (AUCROC). Accuracy refers to the ratio of the number of correctly predicted asymptomatic CAS to the total number of participants [32]. F1 score is composed of a weighted average of precision and recall [33]. Compared to commonly used performance metrics (including recall and specificity), AUCROC better reflected model performance. Hence, AUCROC was the main metric, while accuracy and F1 score were considered as the secondary priorities. Furthermore, a confusion matrix was used to evaluate the performance of the best model.

### Statistical and machine learning analysis

Statistical analysis was performed using SPSS 23.0. Characteristics are presented as mean (± SD) for continuous features and frequencies (%) for categorical features. Binary logistic regression analysis was used to select significant features (*p* < 0.2). Machine learning models were implemented using the Scikit-Learn toolkit in Python version 3.7.4.

## Results

### Data description

We preprocessed 40 continuous features and 19 categorical features, and a total of 19 features were used as input features to develop models (see Additional file 1: Table 2 for more details). Among the 18,441 participants, 6553 were diagnosed with asymptomatic CAS. 59.8% were male, mean age was 50.86 years old and 13.3% were older than 64 years old. Characteristics of the participants are presented in Table 1. The training set consisted of 12,909 subjects (13.4% aged > 64 years old; 59.9% male; 35.5% with asymptomatic CAS), while the testing set consisted of 5532 subjects (13.0% aged > 64 years old; 59.6% male; 35.5% with asymptomatic CAS). In the testing set, hypertension was present in 15.7%, diabetes mellitus in 5.9%, hyperlipidemia in 3.8%, family history in 4.2%, and ever-smoker in 5.1%. All of these values were higher than those in the training set or total participants. The laboratory values of the testing or training sets were similar to those of the total participants. In the subgroup of people over 64 years old, the number of subjects with asymptomatic CAS was significantly higher than that of those without asymptomatic CAS, in both the training and testing sets.

**Table 2:** Comparison of the predictive performance for six models (testing set)

Model	Acc (%)	Sp (%)	Pp (%)	Re (%)	F1 (%)	AUCROC
LR	74.7	86.6	68.6	53.2	59.9	0.809
RF	74.5	89.5	71.3	47.2	56.8	0.794
DT	65.4	71.8	51.2	53.8	52.5	0.628
XGB	73.4	87.8	68.0	47.2	55.7	0.788
GNB	67.0	88.0	63.1	37.2	46.8	0.753
KNN	68.8	81.5	57.7	45.6	50.9	0.704

Acc, accuracy; Sp, specificity; Pp, precision; Re, recall; F1, F1 score; AUCROC, the area under the receiver operating characteristic curve; LR, logistic regression; RF, random forest; DT, decision tree; XGB, eXtreme Gradient Boosting; GNB, Gaussian Naïve Bayes; KNN, K-Nearest Neighbour

### Model comparison for binary classification problem

**Fig. 2:**
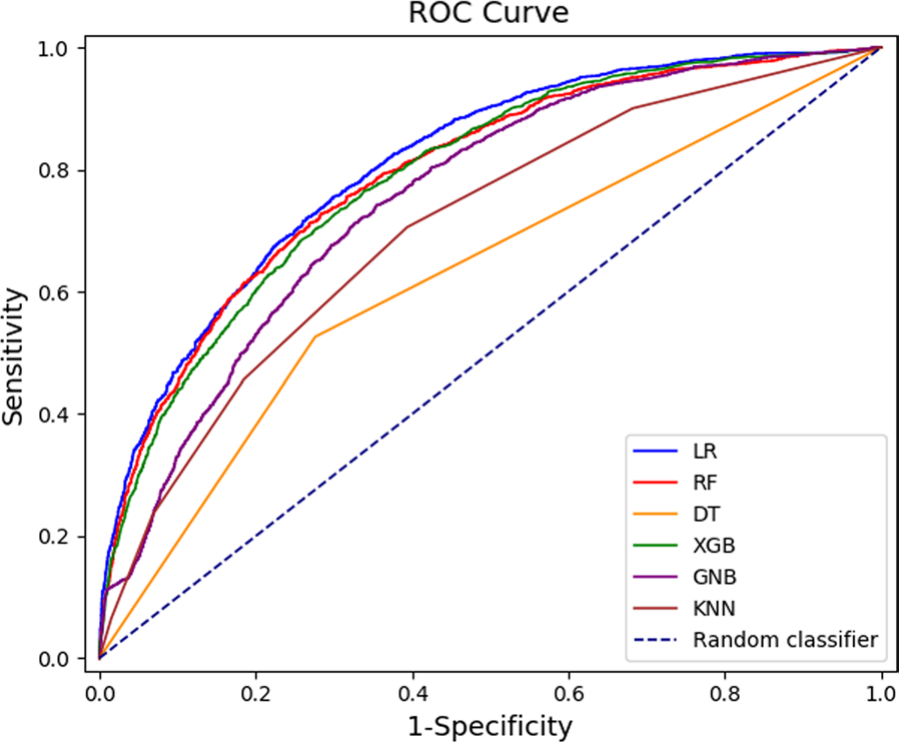
Performance characteristic curves for six models (Testing set). (LR, logistic regression; RF, random forest; DT, decision tree; XGB, eXtreme Gradient Boosting; GNB, Gaussian Naïve Bayes; KNN, K-Nearest Neighbour)

A comparison of the receiver operating characteristic curve for six models is shown in Fig. 2 and Table 2. The differences between these curves were slight, but we could still clearly recognize each model. DT showed the poorest predictive performance, with the lowest AUCROC of 0.628, an accuracy of 65.4%, and a F1 score of 52.5%. In comparison the other five models improved prediction: KNN + 7.6% (AUCROC: 0.704, accuracy: 68.8% and F1 score: 50.9%); GNB + 12.5% (0.753, 67.0%, and 46.8% respectively); XGB + 16.0% (0.788, 73.4%, and 55.7% respectively); RF + 16.6% (0.794, 74.5%, and 56.8% respectively); and LR + 18.1%, which had the highest AUCROC of 0.809, an accuracy of 74.7%, and a F1 score of 59.9%. Detailed predictions of LR were presented in the form of a confusion matrix (see Additional file 1: Table 3 for more details). In the present context, LR was able to predict 1045/1966 asymptomatic CAS (sensitivity 53.2%) and 3088/3566 non-asymptomatic CAS (specificity 86.6%). The results of the tenfold cross-validation showed that LR had a better discriminative ability for asymptomatic CAS than the other five models (Table 3).

**Table 3:** Comparison of the performance for six models (tenfold cross-validation)

Model	AUCROC	Model	AUCROC
LR	0.812	XGB	0.797
RF	0.799	GNB	0.755
DT	0.630	KNN	0.701

**Fig. 3:**
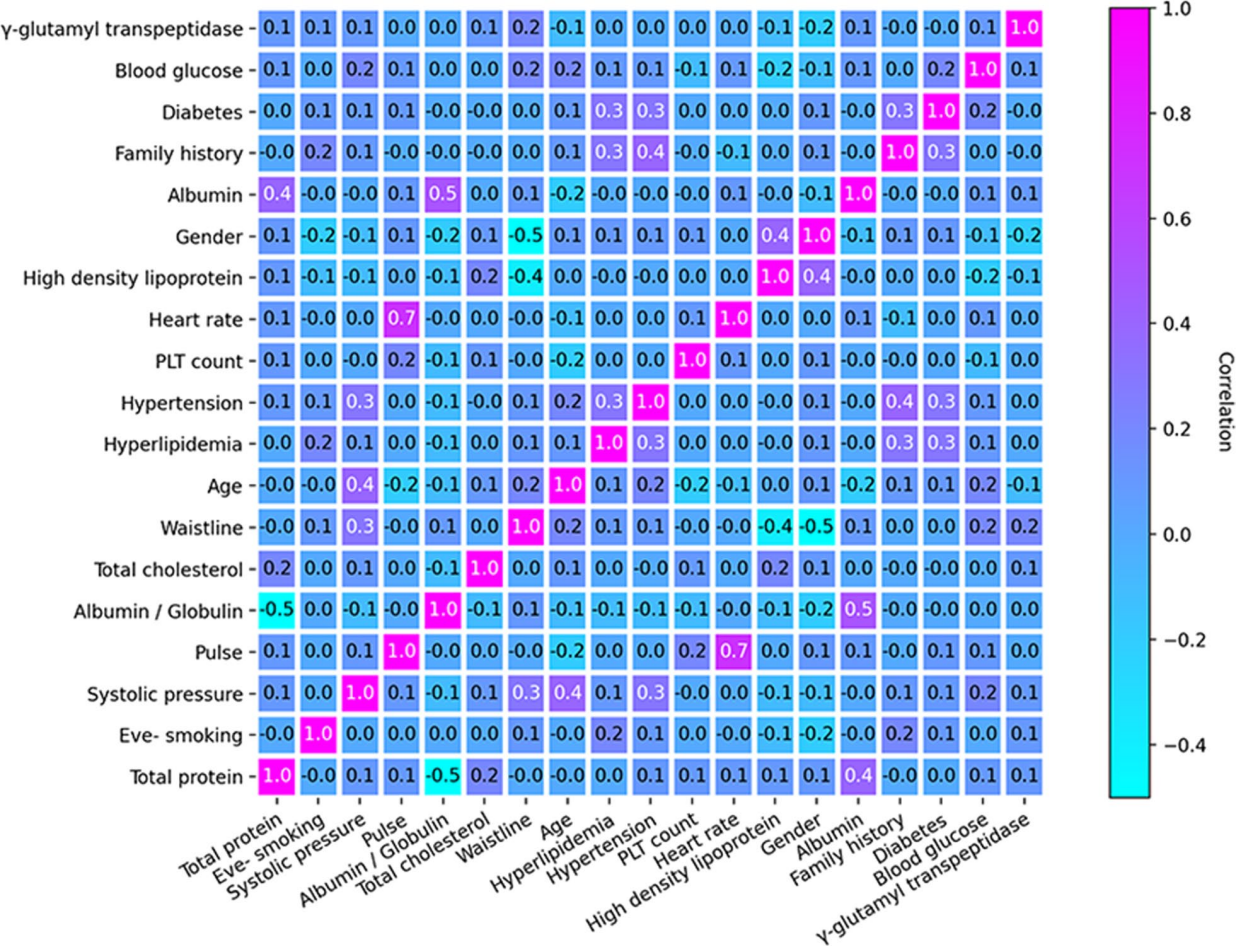
Pearson correlation analysis regarding 19 features for Naïve Bayes. (*PLT*, platelets)

In order to confirm the independence between 19 features, Pearson correlation analysis for NB was performed, as shown in Fig. 3. No significant cross-correlation (Pearson correlation coefficient > 0.8) was observed, which proved that the NB worked well.

**Fig. 4:**
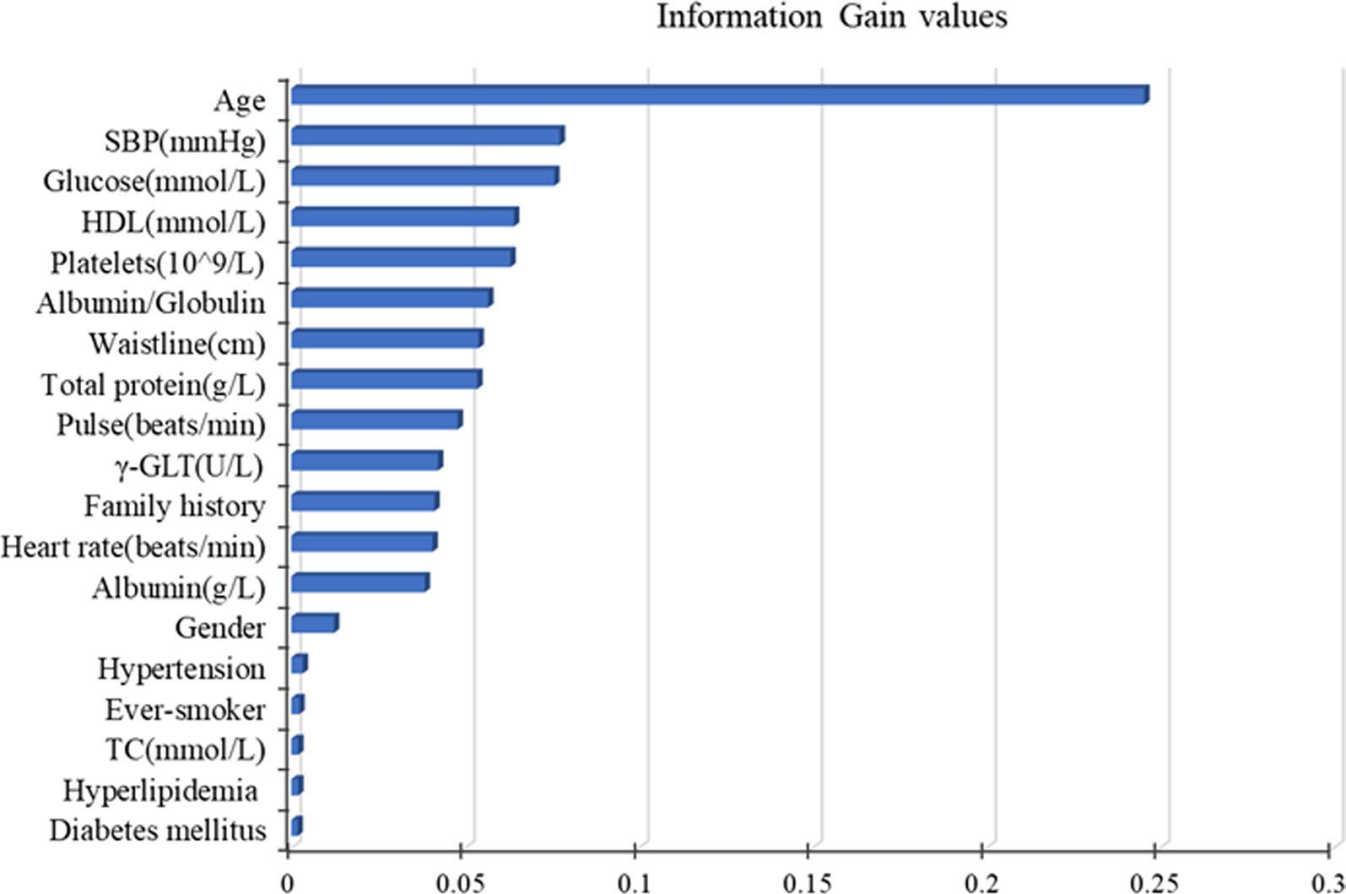
19 features used for decision tree model generation and their information gain values. Features were ranked according to their information gain values which reflect the entropy gain with respect to the predictive outcome. The longer the blue transverse column (the higher the value), the greater importance on the outcome. (SBP, systolic blood pressure; HDL, high density lipoprotein; TC, total cholesterol; γ-GLT, γ-glutamyl transpeptidase)

Moreover, according to the information gain values of DT model, we further ranked those 19 features, as shown in Fig. 4. Age contributed the most to the asymptomatic CAS outcome, followed by systolic blood pressure, glucose, high density lipoprotein, platelets and so on.

## Discussions

In this retrospective analysis, we used six state-of-the-art machine learning models to predict asymptomatic CAS subjects based on 19 input features, which were selected according to binary logistic regression. Among the six models, LR achieved an AUCROC of 0.809, an accuracy of 74.7%, and a F1 score of 59.9% when validating in the separate hold-out testing set, generating the optimal predictive model for data, which was in keeping with other studies using machine learning techniques to assess clinical events [34, 35]. This accuracy was equivalent to or exceeded that of other evaluating tools yet used in neurology [36].

Similar to previous reports, approximately 35.5% of the included subjects presented with asymptomatic CAS. Furthermore, in the subgroup of people over 64 years old, we found that the number of subjects with asymptomatic CAS was significantly higher than that of those without asymptomatic CAS. In accordance with several studies [37, 38], age is one of the robust risk factors of asymptomatic CAS. Population aging poses a threat, and the number of people with CAS will continue to increase, which deserves more attention from the entire society. Perhaps stakeholders should put more effort into intervention measures for this threat in the future.

While machine learning algorithms help us to deal with several problems, they also present an inherent problem increasingly more evident. More often than not, machine learning is still “black box”, lacking sufficient interpretability. It means that researchers or clinicians are progressively relying on the “black box”, achieving results without even knowing what is going on in the machinery of the classifiers. If we had knowledge about some important details about the classifiers, we might achieve one result that could be much more effective. For example, when we used DT, the model already selected the most important features according the information gain values and then split, ranking of the features selected by the DT potentially provided us with additional information. In other words, by focusing on and controlling those high-risk predictors, such as age, systolic blood pressure, and glucose, we would see a more positive tendency throughout the individual's entire CAS treatment. Delaying the progression of CAS would be a tremendous relief for individuals, clinicians, and healthcare systems.

According to complex multidimensional clinical data, Xia Hu, and colleagues [39] constructed a Bayes-based learning framework to reveal predictive insights into the rapid progression of atherosclerosis in prediabetics at risk. And they found that NB was the best, obtaining an AUCROC of < 0.800. Combined with machine learning techniques, Sebastian Okser et al. [40] used the “grey zone” of genetic variation to predict increased risk of pre-clinical CAS. After spending a full 6-year period, they achieved AUCROCs of 0.844 and 0.761 when predicting the extreme classification of CAS risk and progression. The major disadvantages of the above assessment models, lie not only in the specific selection of samples (such as for prediabetics) but also in the requirement of a longer period. Whereas our proposed framework predicted the imminent future for medical check-up subjects, making it a much more pragmatic prescreening tool for clinicians.

Because of binary logistic regression-based feature selection and considerable hyperparameter tuning, machine learning approaches were compared at their best performance. We believe that the input features in the population-based models should depend on the availability of clinical evidence and clinical data, rather than statistical significance, thus feature selection was set at a filter *p* value of 0.2. One of the key results was that the addition of the tenfold cross-validation further verified the superior predictive ability of LR. As a widely used predictive tool in the real-world clinic, LR has extended its potential to improve prediction of precise asymptomatic CAS. Consequently, we envision that the predictions from our models will warn clinicians to pay attention to individuals who are at elevated risk of asymptomatic CAS and make them the major beneficiaries.

Based on MINimum Information for Medical AI Reporting (MINIMAR) [41], there were indeed some limitations to be noted. We believe that the data from multi centers would provide reliable predictive value on how our models identify asymptomatic CAS without selection bias. Due to the limited electronic records available from the “Rocket Frog” system, we did not include the image information, lending our inability to accurately predict the location of atherosclerosis. Moreover, the lack of external validation based on other health systems may limit the generalizability of our models.

## Conclusions

In this study, we demonstrated that the logistic regression model produced a more accurate and effective prediction for asymptomatic CAS among six machine learning models. These findings set the stage for an early automatic alarming system, allowing a more precise allocation of CAS prevention measures to individuals probably to benefit most. Future large-scale studies are needed to provide more reliable and precise data for the prediction of asymptomatic CAS. Using more image information and advanced machine learning schemes are also promising.

## Supplementary Information

**Additional file 1.** Table 1–3: Candidate features, feature selection, and confusion matrix.

## Data Availability

The datasets generated and/or analysed during the current study are not publicly available due privacy but are available from the corresponding author on reasonable request.

## Abbreviations

CAS: Carotid atherosclerosis
XGB: EXtreme Gradient Boosting
GNB: Gaussian Naïve Bayes
KNN: K-Nearest Neighbour
NB: Naïve Bayes
LR: Logistic regression
RF: Random forest
DT: Decision tree
AUCROC: The area under the receiver operating characteristic curve
ACC: Accuracy
F1: F1 score
Sp: Specificity
Pp: Precision
Re: Recall
SBP: Systolic blood pressure
HDL: High density lipoprotein
TC: Total cholesterol
γ-GLT: γ-Glutamyl transpeptidase

## Declarations

### Statement

All machine learning methods were carried out in accordance with the “Guidelines for Developing and Reporting Machine Learning Predictive Models in Biomedical Research”. And the protocol conformed to the Declaration of Helsinki.
