# Supplementary material for: The prediction of asymptomatic carotid atherosclerosis with electronic health records: a comparative study of six machine learning models
Source: BMC Med Inform Decis Mak. 2021 Apr 5;21:115. doi: 10.1186/s12911-021-01480-3 (PMC8020544; doi:10.1186/s12911-021-01480-3)
Supplement: Supplementary file 1 — Additional file 1. Table 1–3: Candidate features, feature selection, and confusion matrix. [file 12911_2021_1480_MOESM1_ESM.docx]

**Additional Table 1** Candidate features for predicting asymptomatic CAS

| Feature group | Feature type | Features |
| --- | --- | --- |
| **Patient characteristics** | Continuous  Categorical | Age, height, weight, BMI, waistline, hipline, and waist to hip ratio  Gender, region |
| **Vital signs** | Continuous | Systolic blood pressure, diastolic blood pressure and heart rate |
| **Co-morbid conditions** | Categorical | Coronary artery disease, diabetes mellitus, prior stroke, hyperlipidemia, hypertension, family history, hepatobiliary disease, kidney disease, respiratory disease, gastrointestinal disorder, other disease, any cancer, surgery, and ever-smoker |
| **Laboratory**  **Values** | Continuous | Alanine aminotransferase, glucose, uric acid, aspartate aminotransferase, creatinine, total bilirubin, direct bilirubin, indirect bilirubin, γ-glutamyl transpeptidase, albumin, globulin, alkaline phosphatase, carcinoembryonic antigen, alpha fetoprotein, total protein, hemoglobin concentration, urea, total cholesterol, high density lipoprotein, low density lipoprotein, white blood cells, red blood cells, hematocrit, lymphocyte percentage, monocyte percentage, neutrophil percentage, albumin/globulin and platelets |
| **Physical examinations** | Continuous  Categorical | Pulse  Heart rhythm, heart murmur and ECG |

(CAS, carotid atherosclerosis; BMI, body mass index; ECG, Electrocardiograph).

**Additional Table 2** Binary logistic regression for feature selection

| Features | B | *Wald* | *p* | OR |
| --- | --- | --- | --- | --- |
| Age | 0.09 | 1456.93 | 0.000 | 1.098 |
| Gender | 0.51 | 45.35 | 0.000 | 1.671 |
| Hypertension | 0.24 | 14.09 | 0.000 | 1.269 |
| Diabetes mellitus | 0.32 | 10.17 | 0.001 | 1.376 |
| Hyperlipidemia | 0.20 | 1.83 | 0.176 | 1.220 |
| Family history | 2.88 | 381.91 | 0.000 | 0.056 |
| Ever-smoker | 0.11 | 1.70 | 0.192 | 0.893 |
| Glucose | 0.07 | 24.93 | 0.000 | 1.076 |
| HDL | 0.33 | 4.14 | 0.038 | 0.719 |
| Total cholesterol | 0.20 | 2.59 | 0.107 | 1.224 |
| Total protein | 0.04 | 4.52 | 0.033 | 1.044 |
| Albumin | 0.08 | 5.87 | 0.015 | 0.927 |
| Albumin/Globulin | 0.77 | 8.72 | 0.003 | 2.163 |
| γ-GLT | 0.00 | 2.36 | 0.124 | 1.001 |
| Platelets | 0.00 | 2.30 | 0.107 | 1.002 |
| Systolic blood pressure | 0.02 | 78.69 | 0.000 | 1.016 |
| Heart rate | 0.01 | 2.78 | 0.095 | 0.995 |
| Pulse | 0.00 | 2.59 | 0.108 | 0.996 |
| Waistline | 0.02 | 1.98 | 0.159 | 1.018 |

(HDL, high density lipoprotein; γ-GLT, γ-glutamyl transpeptidase).

**Additional Table 3** The confusion matrix for logistic regression (Testing set)

| Confusion matrix | Predicted (T) | Predicted (F) |
| --- | --- | --- |
| Actual (T) | 1045 | 921 |
| Actual (F) | 478 | 3088 |
